# High Resolution pH Measurements Using a Lab-on-Chip Sensor in Surface Waters of Northwest European Shelf Seas

**DOI:** 10.3390/s18082622

**Published:** 2018-08-10

**Authors:** Victoire M. C. Rérolle, Eric P. Achterberg, Mariana Ribas-Ribas, Vassilis Kitidis, Ian Brown, Dorothee C. E. Bakker, Gareth A. Lee, Matthew C. Mowlem

**Affiliations:** 1 National Oceanography Centre, Southampton, University of Southampton, Southampton SO14 3ZH, UK; v.rerolle@fluidion.com (V.M.C.R.); mariana.ribas.ribas@uni-oldenburg.de (M.R.-R.);; 2 GEOMAR Helmholtz Centre for Ocean Research Kiel, 24148 Kiel, Germany; 3 Institute for Chemistry and Biology of the Marine Environment, University of Oldenburg, 26382 Wilhelmshaven, Germany; 4 Plymouth Marine Laboratory, Prospect Place, Plymouth PL1 3DH, UK; vak@pml.ac.uk (V.K.); iaian2@pml.ac.uk (I.B.); 5 Centre of Ocean and Atmospheric Sciences, School of Environmental Sciences, University of East Anglia, Norwich NR4 7TJ, UK; d.bakker@uea.ac.uk (D.C.E.B.); g.a.lee@uea.ac.uk (G.A.L.); 6 National Oceanography Centre, Southampton SO14 3ZH, UK; matm@noc.ac.uk

**Keywords:** seawater pH, lab-on-chip, LOC, spectrophotometry, microfluidics, European shelf seas, high resolution pH measurements

## Abstract

Increasing atmospheric CO_2_ concentrations are resulting in a reduction in seawater pH, with potential detrimental consequences for marine organisms. Improved efforts are required to monitor the anthropogenically driven pH decrease in the context of natural pH variations. We present here a high resolution surface water pH data set obtained in summer 2011 in North West European Shelf Seas. The aim of our paper is to demonstrate the successful deployment of the pH sensor, and discuss the carbonate chemistry dynamics of surface waters of Northwest European Shelf Seas using pH and ancillary data. The pH measurements were undertaken using spectrophotometry with a Lab-on-Chip pH sensor connected to the underway seawater supply of the ship. The main processes controlling the pH distribution along the ship’s transect, and their relative importance, were determined using a statistical approach. The pH sensor allowed 10 measurements h^−1^ with a precision of 0.001 pH units and a good agreement with pH calculated from a pair of discretely sampled carbonate variables dissolved inorganic carbon (DIC), total alkalinity (TA) and partial pressure of CO_2_ (pCO_2_) (e.g., pH_DICpCO2_). For this summer cruise, the biological activity formed the main control on the pH distribution along the cruise transect. This study highlights the importance of high quality and high resolution pH measurements for the assessment of carbonate chemistry dynamics in marine waters.

## 1. Introduction

About a quarter of the emitted anthropogenic CO_2_ is taken up by the oceans [1], resulting in a decrease in seawater pH [2]. The capacity of the oceans to sequester CO_2_ depends on the roles of biological (soft tissue and carbonate) and solubility pumps in altering CO_2_ concentrations in the surface ocean [3]. Coastal seas are potentially a significant sink of CO_2_ (0.2 Pg C yr^−1^) due to a high biological activity and a net off shelf carbon transport at depth to the adjacent deep ocean regions [4,5,6]. Coastal environments are highly dynamic, subject to interacting biological and physical processes with contrasting effects on the marine carbonate system and therefore on the capacity of coastal seas to absorb CO_2_.

The spatial and temporal resolution of marine carbonate system measurements is currently insufficient, despite important efforts to improve analytical methods, develop new instruments and coordinate international monitoring activities [7,8]. Instruments measuring carbonate variables need to be automated, miniaturized and ruggedized to be deployed on ships of opportunity and in situ platforms such as moorings, gliders, profiling floats. Widespread use as part of a global observing system, will only be possible with low-cost sensors featuring low resource consumption. Miniaturization of analytical systems is leading to a reduction in sample, reagent and power consumption. Wet chemical sensors utilise similar chemistries to standard laboratory techniques, providing advantages in terms of sensitivity, analyte specificity, traceability and reliability. A key advance has been the implementation of microfluidic Lab-on-Chip platforms for wet chemical analyses, and this technology is of great interest for environmental monitoring in marine systems [9,10].

pH is one of the four variables of the oceanic carbonate system that can be routinely and reliably analysed (the others are dissolved inorganic carbon (DIC), total alkalinity (TA) and partial pressure of CO_2_ (pCO_2_)). The widely-used spectrophotometric pH technique delivers high-quality measurements that are required to study the changes in the marine carbonate system [11]. High precision and accuracy pH measurements using the spectrophotometric technique have been demonstrated at sea for surface water measurements on research vessels [12,13,14,15,16,17,18,19,20,21], and successful in situ deployments have been reported [22,23,24,25]. The spectrophotometric method is calibration-free [11], which is ideal for long-term deployments for pH measurements on ships and remote platforms. This simple and high precision technique has recently been successfully miniaturized on a Lab-on-Chip platform [26] as a first step towards autonomous deployments. This technology was deployed on-board a research vessel in the current study, forming a first unique demonstration of its capability.

Alternative pH measurement approaches for seawater include the widely employed electrochemical techniques. The ion-sensitive field-effect transistor (ISFET) sensors utilise advanced electrochemical technologies [23] with availability of commercial sensors, but they are subject to drift and hence require regular calibration. The fluorescent optode pH technique [27] forms a promising approach for seawater measurements but is still in its infancy with no commercial systems available yet.

The main factors controlling the carbonate chemistry in coastal waters are biological activity, temperature changes and discharges of river water [28]. The photosynthetic uptake of CO_2_ to form organic matter (biological production) can be expressed as [29]:

106CO_2_ + 16NO_3_^−^ + HPO_4_^2−^ + 78H_2_O + 18H^+^ = C_106_H_175_O_42_N_16_P + 150O_2_ (1)

The nitrate uptake to form organic nitrogen (e.g., C_106_H_175_O_42_N_16_P) results in an increase in TA. The formation of organic matter therefore not only decreases DIC but also increases TA proportionally to the ratio of carbon to nitrogen. Biological production therefore increases seawater pH, whereas respiration and remineralisation of organic matter lead to a decrease in pH.

Temperature changes affect the carbonate equilibrium constants as detailed in the following expressions of the dissociation constants K_1_ and K_2_ for carbonic acid from [30] as refitted by Dickson and Millero [31]:

log_10_(K_1_) = −62.008 + 3670.7/T + 9.7944ln(T) − 0.0118S + 0.000116S^2^ (2)

log_10_(K_2_) = 4.777 + 1394.7/T − 0.0184S + 0.000118S^2^ (3)

An increase in temperature increases the carbonate equilibrium constants and results in a decrease in pH [32] (see Equation (1)) and CO_2_ solubility [33]. However an increase in temperature will typically also enhance water column stratification and primary productivity [34], increasing surface water pH.

Riverine inputs can have differing effects on pH. Rivers can result in direct changes in coastal carbonate chemistry through the discharge of high DIC and low TA freshwaters [28,35,36], thereby lowering pH. Additionally, nutrient supply by rivers can stimulate primary productivity in coastal waters, thereby increasing pH, whereas respiration of supplied riverine organic matter will decrease pH [37]. Localised upwelling at shelf breaks of relatively acidic DIC-rich deep waters will also significantly decrease pH while simultaneously enhancing primary productivity through nutrient supply [38,39]. Finally, surface water uptake of anthropogenic atmospheric CO_2_, and deposition of HNO_3_ and H_2_SO_4_ aerosols will decrease seawater pH [40].

The strong spatial and temporal variability in the surface water carbonate chemistry has led to contradicting estimates of air-sea CO_2_ fluxes in coastal seas. Most of the processes affecting the carbonate system are usually occurring simultaneously, making it challenging to understand and determine the main drivers of the dynamics of the carbonate chemistry in coastal seas. This highlights the need for datasets with high resolution to unravel the various processes and their consequences for the carbonate system.

The carbonate chemistry in the English Channel and the North Sea has been well studied and particularly air-sea CO_2_ fluxes using pCO_2_ data are widely reported [41,42], however carbonate chemistry data is lacking for other parts of the Northwest European shelf seas.

We present here a new data set of surface water pH determined in summer 2011 in the North West European Shelf Seas, one of the largest continental shelf systems on earth. It is the first time that pH has been measured at a high spatial resolution (10 measurements h^−1^) in this shelf seas region. A Lab-on-Chip sensor was used to determine pH, utilising a spectrophotometric approach with Thymol Blue as indicator dye [26]. The aim of our paper is to report on the use of the novel Lab-On-Chip pH sensor and investigate the dynamics in carbonate chemistry of the shelf seas surface waters using pH and ancillary data. The unique high resolution pH data set allowed us to use statistical approaches to investigate the processes controlling pH and their relative importance in explaining the observed pH variance along the ship’s transect. We explain pH dynamics using solely underway pH, temperature, salinity, and fluorescence data. The data interpretation is supported by lower resolution surface water nutrient data, in addition to variables determined through discrete sampling at CTD stations (e.g., dissolved organic carbon (DOC), TA, DIC).

## 2. Method

### 2.1. Cruise

**Figure 1:**
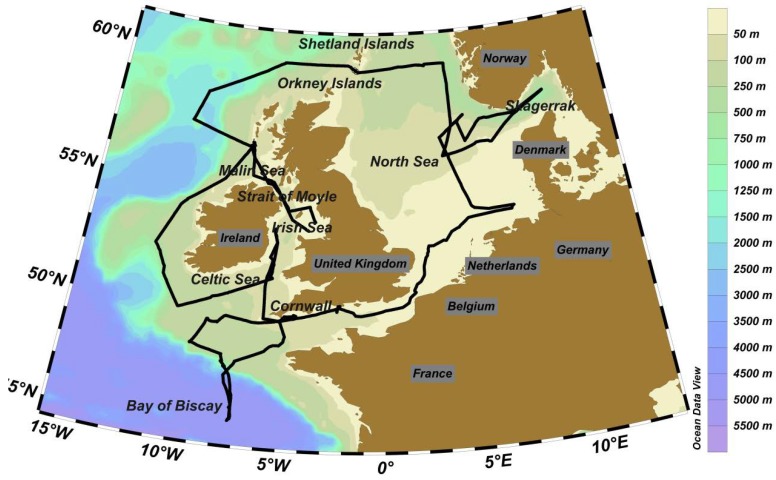
Map of the D366 cruise track (black line) with bathymetry contours (colour scale). Map was produced using Ocean Data View (Schlitzer, R., Ocean Data View, odv.awi.de, 2017).

The data used in this study have been collected in the period between 6 June 2011 and 7 July 2011 during the RRS *Discovery* research cruise D366 in Northwest European shelf waters (see cruise track in Figure 1). The four variables of the carbonate system (DIC, TA, pCO_2_ and pH) were determined at a high spatial resolution (DIC and TA every hour, pCO_2_ and pH every 6 min with a typical speed of the vessel of 10 knots) thereby over-constraining the carbonate system and facilitating internal data quality verification [43].

### 2.2. Data

#### 2.2.1. Underway Measurements

**Figure 2:**
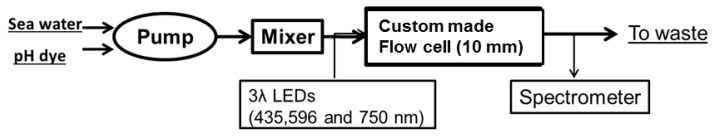
Schematic of the Lab-on-Chip pH analyser. The microfluidic flow cell comprised of an absorption cell and static mixer produced in poly(methyl methacrylate) (PMMA) [45]. Two syringe pumps (Harvard Apparatus Nanomite, Kent, UK) and four micro-inert valves (LFNA1250125H, Lee Products Ltd., Gerrards Cross, UK) controlled the fluidics and were directly mounted on the chip. The 10 mm absorption cell was connected to the light source and detector by two optical fibers (600 µm diameter, Thorlabs Inc., Newton, NJ, USA), and had a volume of 5 µL. A tri-colored LED was used as lightsource, with wavelengths: 435 nm and 596 nm corresponding to the absorption maximum of the Thymol Blue indicator forms HI^−^ and I^2−^, and 750 nm to monitor turbidity of the sample. A linear array photodiode spectrophotometer (HR4000, Ocean Optics, Oxford, UK) was used as detector.

Surface water pH was measured continuously with an automated sensor connected to the ship’s underway water supply, which has an intake at approximately 5 m depth, and placed in the ship’s laboratory. To avoid absorbance interference by particulate matter, an in-line filter (0.45 μm pore size, Millex HP PES, Millipore, Merck, Darmstadt, Germany) was placed on the sample tube. The automated pH system was operated continuously in the period between 6 June 2011 and 7 July 2011. The pH measurements were conducted following the colorimetric method from Clayton and Byrne using Thymol Blue (ACS Reagent, Sigma Aldrich, Merck, Darmstadt, Germany) as pH indicator [11,44]. Figure 2 presents a schematic of the Lab-on-Chip sensor, full details are provided in [26].

The pH of a sample is determined from the acid dissociation constant (pK_ind_ = −log_10_(K_HI−_)) and the absorbance ratio of the deprotonated (I^2−^) and protonated (HI^−^) Thymol Blue indicator forms using the following equation [46]:

pH = pK_ind_ + log_10_((R − e_1_)/(e_2_ − Re_3_)) (4)

with R = A_1_/A_2_, e_1_ = ε_1_(HI^−^)/ε_2_(HI^−^), e_2_ = ε_1_(I^2−^)/ε_2_(HI^−^) and e_3_ = ε_2_(I^2−^)/ε_2_(HI^−^)). A_1_ and A_2_ are the absorbances at wavelength 1 and 2. ε_λ_(I^2−^) and ε_λ_(HI^−^) refer to the molar absorptivity coefficients at wavelength λ (with subscripts 1 and 2 corresponding to the wavelengths at maximum absorptivity of the basic and acidic forms of the indicator, respectively) of the base (I^2−^) and acid (HI^−^) forms of the dye, respectively. The molar absorptivity coefficients and the pK_ind_ are functions of temperature and salinity.

A total of 29,950 pH measurements were conducted with the Lab-on-Chip sensor in surface waters along the cruise track. The pH measurements were made on the total pH scale (pH_tot_), at a frequency of 10 measurements h^−1^ and with a precision of 0.001 pH units. Three bottles of Tris pH buffer (certified on the pH_tot_ scale), provided by Marine Physics Laboratory of Scripps Institute of Oceanography, University of San Diego were analysed at the start, middle and end of the cruise to verify the accuracy of the pH_tot_ measurements, which was 0.004 pH. Chromophoric dissolved organic matter absorbs weakly in the visible spectrum where Thymol Blue absorbance is measured (<3% at 435 nm and <1% at 596 nm), and was accounted for using the measurement of a seawater blank (conducted for every sample analysis). Measurements at a wavelength 750 nm (not affected by the indicator Thymol Blue) were employed to monitor for instrument drift and sample turbidity and the data were corrected accordingly.

The indicator extinction coefficients of Thymol Blue were determined after the cruise on the pH instrument and for the salinity and temperature ranges observed during the cruise [26], and a reported Thymol Blue pK_2_ was used [47].

The seawater temperature increased by 0.2 °C from the ship’s intake to the pH instruments and pH was corrected accordingly to in situ temperature using the DIC data [32]. For this purpose, the pH and DIC data were used to calculate TA. DIC and TA are not temperature dependent and can therefore be used to calculate pH at the in situ oceanic temperature using thermodynamic relationships. A linear relationship between pH and temperature was derived from this correction and used to calculate pH at in situ temperature where no DIC value was available. The magnitude of the correction was about 0.002 ± 0.001 pH units. An additional correction of +0.004 pH units has been applied to the pH data to correct for an analysis perturbation due to the Schlieren effect [26].

pCO_2_ measurements in surface waters (from ship’s underway supply) were made using a system with a shower-head equilibrator and an infrared gas analyser (LICOR, LI-840) [48], and referenced against standard CO_2_ gases. The precision of the pCO_2_ measurements was 1 μatm, with an accuracy of 4 μatm [43]. Continuous sea surface temperature (SST), conductivity/salinity (SSS) and chlorophyll-*a* fluorescence (Chl) data were obtained from a Sea-Bird Electronics SBE45 ThermoSalinoGraph (TSG) installed on the ship’s underway supply.

#### 2.2.2. Discrete Underway Water Samples

Discrete seawater samples for DIC, TA and nutrients were collected every two hours from the ship’s underway supply. Discrete water samples for SSS were also collected every four hours in order to calibrate the underway conductivity measurements. Discrete salinity samples were analysed using a salinometer (Guildline Autosal 8400B, Sorrento, FL, USA).

The DIC and TA samples were collected using standard protocols [49] in 250 mL glass bottles, poisoned with a saturated mercuric chloride solution, and subsequently analysed for DIC and TA using a VINDTA 3C instrument (Marianda, Kiel, Germany) [50]. Measurements were calibrated using certified reference material from Prof. Dickson, Scripps (159 CRMs analysed, in duplicate, over duration of cruise). The accuracy of the DIC and TA measurements was 2.0 and 1.5 μmol kg^−1^, and precision 1.7 and 1.2 μmol kg^−1^, respectively.

Analysis of nitrate and nitrite (total oxidised nitrogen, TON), phosphate (PO_4_^3−^) and silicic acid (SiO_4_^4−^) were undertaken using a segmented flow auto-analyser (Skalar San+, Breda, The Netherlands) following methods described by Kirkwood [51]. Samples were stored in 25 mL polycarbonate vials and kept refrigerated at approximately 4 °C prior to the nutrient analysis (within 12 h after sampling).

#### 2.2.3. CTD Variables

Depth sampling in the water column along the cruise track was undertaken using a standard stainless steel CTD rosette system and Niskin bottles. The dissolved oxygen (O_2_) sensor of the CTD was calibrated using oxygen measurements on discretely collected samples using automated Winkler titration with photometric endpoint detection [52]. Samples for DOC were collected using the Niskin bottles, filtered using ashed glass fibber filters (Whatman GF/F) and analysed using a high temperature combustion technique [53].

#### 2.2.4. Quality Control of Carbonate Chemistry Data

A comprehensive quality check and comparison between the different datasets of carbonate variables can be found in [43]. A good agreement was found between all datasets. Comparison of measured pH with pH calculated from a pair of the carbonate variables DIC, TA and pCO_2_ (e.g., pH_DICpCO2_) showed an absolute mean discrepancy between 0.008 and 0.010 pH units, so we conclude that there was good agreement between calculated and measured pH.

### 2.3. Study Region: Hydrography of the Northwest European shelf seas

**Figure 3:**
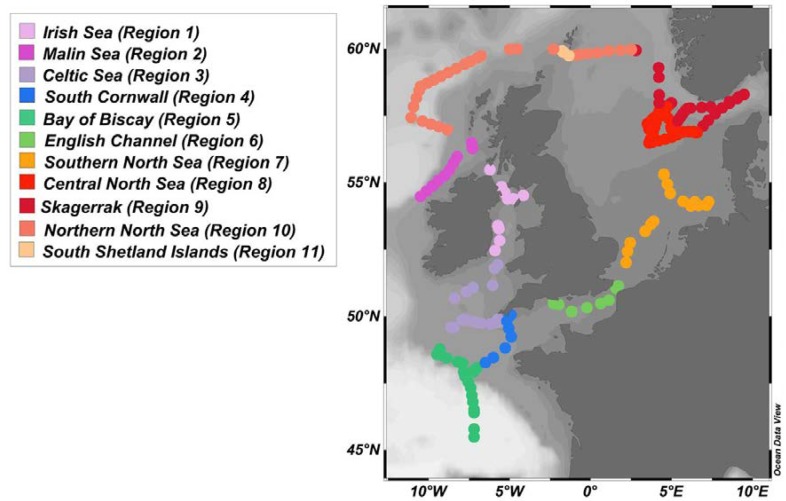
The 11 regions indicated using colour coding and defined using geographical and water mass (SST-SSS) characteristics. Map was produced using Ocean Data View (Schlitzer, R., Ocean Data View, odv.awi.de, 2017).

The Northwest European Shelf Seas are typically shallower than 250 m with the exception of the deep Norwegian Trench [54] (Figure 3). In the Skagerrak Strait depths reach 700 m. The southern North Sea is shallower than 50 m and increases in depth towards the north to ca. 150 m. The northern North Sea is seasonally stratified [28], whereas the shallow southern North Sea is continuously mixed throughout the year receiving the majority of the riverine fresh water inputs to the North Sea. The Malin Sea on the shelf edge to the north of Ireland also becomes seasonally stratified [55]. The Celtic Sea and the English Channel increase in depth towards the shelf edge with the Atlantic Ocean, with the shallow waters in the eastern English Channel being mixed throughout the year, and the deeper western English Channel and Celtic Sea waters exhibit stratification in summer [56,57]. The Irish Sea receives large freshwater inputs, and communicates with the Atlantic Ocean via the Malin Sea to the northwest and the Celtic Sea to the southwest. The Bay of Biscay is situated on the southern extend of the Northwest European shelf system and features deep oligotrophic and seasonally stratified waters [58].

The current system in the North Sea is dominated by an anti-clockwise flow along the edges, with Atlantic waters coming mainly from the northwest opening, and leaving along the Norwegian coast via the Norwegian Trench [59]. Tidal currents can be stronger than the residual current in many areas, particularly shallow regions, and cause important mixing in the water column. The study area is further influenced by important freshwater inputs, and the annual freshwater river input into for example the North Sea is on the order of 300 km^3^ [59]. In addition, the vast amount of brackish waters of the adjacent Baltic Sea is a dominant fresh water source to the North Sea and discharged through the Skagerrak Strait.

The study area has been split into eleven regions which were defined by hydrodynamic characteristics of water masses and stratification (Figure 3), using the regions defined for reporting under the EU Marine Strategy Framework Directive and reports by [42,60], with additions of the Bay of Biscay, Central North Sea and Skagerrak regions. A MANOVA was performed with pH, SST and SSS to determine whether the data from the regions were statistically different.

The Multivariate Analysis Method was used to verify whether the multivariate sample means (here the samples are the regions), are statistically different using pH, T and S. The regions 4 and 11 were relatively small and corresponded to areas where deep water had been locally brought to the surface by storm or shelf mixing. No statistical analysis has been performed with the data from these regions.

### 2.4. Statistical Approach

In order to perform statistical analyses on the data, all the variables have been transformed (using log 10) to obtain a normal distribution and standardised (centred and scaled to 1). Distributions of TON and PO_4_^3−^ were strongly positively skewed, even after transformation. No treatment was done on skewed TON data and this dataset was only used to obtain the sign of the correlation. The data analysis has been performed using the software MatLab^®^.

A stepwise multi-linear regression to relate pH data to environmental variables has first been performed with all the data from the surface samples of CTD stations using the following variables: DOC, O_2_, SSS, SST, Chl, SiO_4_ and TON. The same stepwise multi-linear regression has been performed again but without O_2_. The multiple regression was used to assess whether variables were correlated in order to explore processes which explain the pH distribution. The strong correlation between O_2_ and pH (see Section 3.3.1) was mainly due to the fact that these parameters are affected by similar processes. Hence the analysis is performed again without O_2_ to evaluate what variables correlate with pH.

In addition, multi-linear regressions have been performed to relate the pH data in each individual region with the underway variables SSS, SST and Chl. Finally, stepwise multi-linear regressions have been performed with the underway variables SSS, SST, Chl, SiO_4_ and TON which were obtained at a lower spatial resolution.

## 3. Results and Discussion

### 3.1. Non-Carbonate Data Distributions

Mean values and standard deviations per region for SSS, SST, TON, SiO_4_ and Chl are presented in Table 1. Salinity varied between 33.2 and 35.8 along the cruise transect, apart from the Skagerrak area where salinity was as low as 26.4. Lowest salinities due to riverine inputs were observed in the Irish Sea (region 1), the southern North Sea (region 7) and the Skagerrak area (region 9). Temperature varied between 10.3 and 17.1 °C with lowest values observed at the start of the cruise around Ireland (regions 1 and 2) and in region 11. Highest temperatures were observed in the southernmost part of the cruise transect in the Bay of Biscay (region 5), the southern North Sea (region 7) and the Skagerrak area (region 9). TON and SiO_4_ varied between 0.1 and 5.34 μM with lowest concentrations observed south of Ireland (region 3) and in the northern part of the transect (regions 8, 9 and 10). Highest nutrient concentrations were observed in regions 1, 2 and 11 due to riverine inputs and deep water mixing (region 11, see Section 3.4.4). Chl levels varied between 0.12 and 1.54 µg L^−1^. Highest levels of chlorophyll were observed in the north-west part of the transect in regions 2 and 10, whereas the lowest levels were observed in regions 8 and 9.

### 3.2. Distribution of Carbonate Chemistry Variables

**Figure 4:**
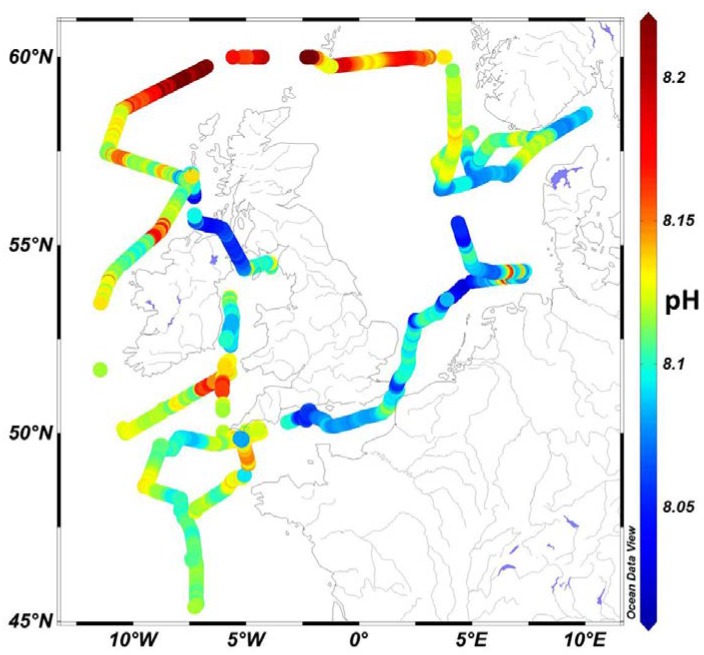
Map of surface water pH_tot_ in European shelf waters for cruise D366 with colour bar indicating pH_tot_ values. Map was produced using Ocean Data View (Schlitzer, R., Ocean Data View, odv.awi.de, 2017).

**Figure 5:**
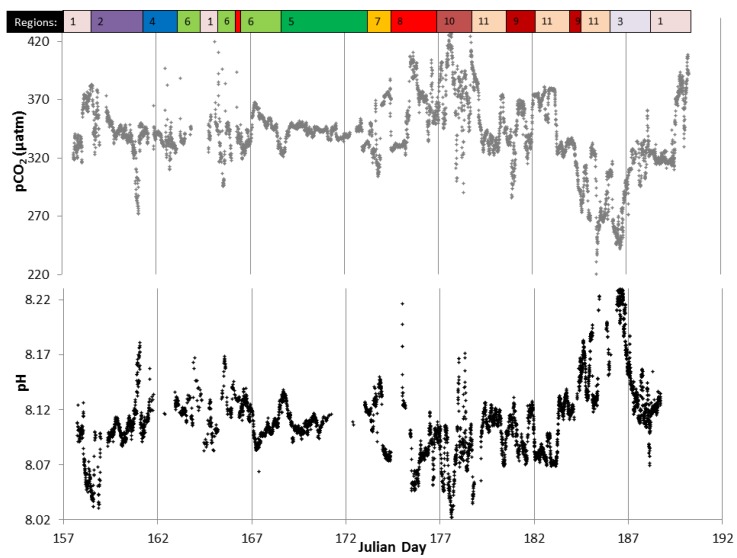
Sea surface pCO_2_ and pH_tot_ for the cruise D366 with the variables plotted against Julian Day. Irish Sea (region 1), Malin Sea; northwest of Ireland (region 2), Celtic Sea (region 3), south of Cornwall, UK (region 4), Bay of Biscay (region 5), English Channel (region 6), Southern North Sea (region 7), Central North Sea (region 8), Skagerrak (region 9), Northern North Sea (region 10), South of the Shetland Islands (region 11).

**Figure 6:**
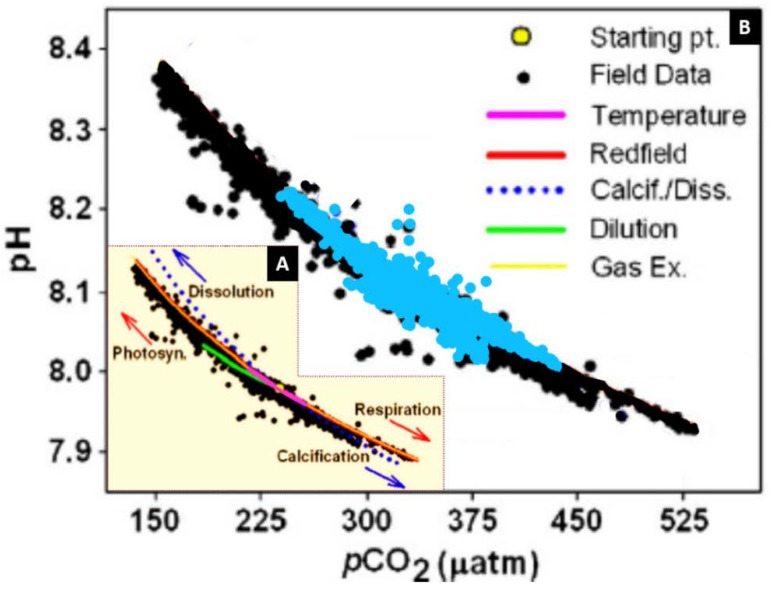
(**A**) Influence on the pH-pCO_2_ relationship of various modelled processes. (**B**) pH versus pCO_2_ for the D366 research cruise (blue dots) plotted over data reported by [64] and collected in Monterey Bay on the MBARI M0 buoy in summer 2007 (black dots). For biological production and respiration (red curve), DIC and TA were varied by the Redfield ratio 106:18 over the range TA = 2243–2289 μmol kg^−1^ and DIC = 1824–2094 μmol kg^−1^ (T = 14.0 °C). Gas exchange (yellow curve) was calculated using a similar range for DIC, but keeping the TA constant (T = 14.0 °C). For calcification and dissolution (blue filled circles), DIC and TA were varied by 1:2 over the range TA = 2090–2770 μmol kg^−1^ and DIC = 1950–2290 μmol kg^−1^ (T = 14.0 °C). For changes in temperature (pink curve), DIC and TA were fixed at 2032 μmol kg^−1^ and 2254 μmol kg^−1^, respectively, and temperature was varied over the range of the field data (11–16 °C). Dilution evaporation/precipitation green curve) was modelled by varying TA and DIC in a 1:1 ratio over the range TA = 1985–2254 μmol kg^−1^ and DIC = 1763–2032 μmol kg^−1^. All calculations were centred on the initial conditions, S = 33.7, DIC = 2032 μmol kg^−1^ and TA = 2254 μmol kg^−1^ (yellow filled circle). Figure adapted from [64].

Surface seawater pH along the transect sampled during early summer (June) ranged between 7.995 and 8.21 (Figure 4 and Figure 5), with highest values in the Northern North Sea featuring enhanced Chl concentrations (up to 1.6 µg L^−1^) and lower temperatures (SSTmean_region10_ = 1.5 ± 0.3 °C). Lowest pH values were observed in the Central North Sea in a well-mixed water column with enhanced DOC concentrations (up to 90 µM compared with 65 µM in the Northern North Sea) and associated enhanced organic matter respiration with a consequent decrease in pH. These observations agree well with reported values ranging from nearly pH_tot_ 8.20 in the Northern North Sea to pH_tot_ 8.05 in the Southern North Sea [61]. Mixed waters in the eastern English Channel also exhibited relatively low pH values (8.07–8.1), with surface pH above 8.1 in the stratified waters of the western English Channel, Celtic Sea, Bay of Biscay, and Malin Sea. Surface pCO_2_ data varied between 220 and 436 µatm, with pH and pCO_2_ variations during the cruise presented in Figure 5. Atmospheric pCO_2_ in June 2011 was ca. 396 μatm at the Mace Head Atmospheric Research Station (Western Ireland), and therefore along most of the study region the waters were undersaturated with respect to atmospheric CO_2_ and formed a carbon sink. Waters in the English Channel and Bay of Biscay are reported to be a CO_2_ sink during summer periods [62], with similar observations by [63] for waters corresponding to our region 2 (average pCO_2_ 345 μatm, in June 2009). Waters in parts of the Irish Sea (region 1), south of Cornwall, UK (region 4), English Channel (region 6), Central North Sea (region 8), and Northern North Sea (region 10) showed higher pCO_2_ compared with atmospheric values, and hence constituted a carbon source to the atmosphere. pH displayed a typical anti-correlation with pCO_2_ (Figure 6).

The pH-pCO_2_ variations were controlled by temperature variations, biological activity, calcification, gas exchange and dilution processes (e.g., freshwater inputs). The impacts of these processes on the pH-pCO_2_ relationship are illustrated in Figure 6A. The pH and pCO_2_ data measured in the North West European Shelf Seas in summer 2011 agreed well with the range of these variables observed in Monterey Bay in summer 2007 (Figure 6B). The relationship between pCO_2_ and hydrogen ions concentration can be expressed as:

DIC ≈ K_1_K_0_pCO_2_/[H^+^] (5)

with K_0_ and K_1_ representing the CO_2_ solubility and first dissociation constant for carbonic acid, respectively [64]. This relationship indicates that processes changing the seawater carbonate chemistry will have opposite impacts on pH and pCO_2_. An increase in DIC or in the carbonate constants (K_1_ and K_2_) will result in an increase in pCO_2_ and a decrease in pH.

### 3.3. pH Control by Environmental Forcings

#### 3.3.1. Surface Data from CTD Stations along the Full Transect

The pH data were strongly correlated with O_2_ for the surface samples from the CTD stations along the full transect (correlation coefficient Rho = 0.5987, *p* = 1.3815 × 10^−7^, *n* = 62). The stepwise multi-linear regression with DOC, O_2_, S, T, Chl, SiO_4_ and TON indicated that the correlation of O_2_, SST and SSS with pH was statistically significant and explained 72% of the pH variance. O_2_, SST and SSS were found to explain 37%, 24% and 11%, respectively, of the variance in pH. The strong correlation with O_2_ indicated that pH and O_2_ were affected by similar processes, with primary productivity and remineralisation of organic matter playing a key role in the pH variations along the transect. The same stepwise multi-linear regression but without O_2_ resulted in SiO_4_ (18%), DOC (15%) and Chl (11%) explaining 44% of the pH distribution along the cruise transect.

#### 3.3.2. Underway Data for the 11 Regions

The statistical analysis, undertaken using underway data, further explains the extent to which the pH variance is explained by the various forcings (Table 2). The large amount of underway data (29,500 data points for pH), allowed a thorough statistical data interrogation, which would not have been possible with datasets obtained using usual shipboard sampling of CTD stations. The pH variability along the transect was strongly determined by biological activity as indicated by strong positive contributions of Chl to the pH variance. Biological processes were the dominant control over pH in regions 1 (Irish Sea), 2 (Malin Sea), 3 (Celtic Sea), 5 (Bay of Biscay) and 10 (Northern North Sea), with all except region 1 exhibiting water column stratification. Whilst the relative contribution of Chl to pH variance is high for region 7 (Southern North Sea), only a small part of the pH variance is expressed by the regression analysis (21%; see further discussion below). Temperature was dominant (highlighted with strong negative correlations) in regions 8 (Central North Sea) and 9 (Skagerrak), with still an important contribution by biology on pH in region 8. Regions 6 to 9 (English Channel, Southern North Sea, Central North Sea and Skagerrak) showed a different pattern, where mixing and outflows from rivers and the Baltic Sea had a strong impact on the carbonate chemistry, as also indicated by the elevated pCO_2_ in parts of the regions (Figure 5). Overall between 21 and 79% of the pH variance can be explained using only salinity, temperature and chlorophyll. The lowest percentages were obtained in regions where riverine inputs impacted the pH distribution (regions 3 (Celtic Sea), 6 (English Channel) and 7 (Southern North Sea)). The impact of freshwater inputs by rivers is evidenced by enhanced correlations between pH and salinity, but the sign of the correlation will depend on the alkalinity (and DIC) levels in the river discharges. A positive correlation between pH and nutrients, and a negative correlation with dissolved organic matter can be further indications of riverine inputs supplying enhanced nutrient and DOC fluxes to coastal waters. The low R^2^ obtained in region 10 was due to the fact that the impact of primary production was not directly evidenced by a correlation of chlorophyll concentration with pH (see section below).

The results of the statistical approach helped to untangle some of the key processes controlling the pH distribution in the different regions of the dynamic shelf system. A limitation of our sampling approach is that the samples were collected at various times of the day, which will influence surface water pH, particularly in areas with pronounced phytoplankton blooms [41].

Our observations are in agreement with previous studies investigating the controls on surface water pCO_2_ in the North Sea [28] and the English Channel [36]. Indeed, Thomas and Salt and co-workers observed that biological activity was the main driver of the seasonal pCO_2_ variability in the northern and central part of the North Sea, whereas temperature variations were driving the pCO_2_ variability in the southern part. The fully mixed waters of the southern part were a source of CO_2_ to the atmosphere, but not in spring when primary production was more important in determining pCO_2_ levels than temperature increases. During our cruise in June, the surface water and atmospheric pCO_2_ were near equal in our region 7 (Southern North Sea) (Figure 5). Similarly, Gypens and co-workers observed a seasonal and inter-annual antagonism between biological and temperature effects on pCO_2_ (and pH) in the English Channel and the Southern Bight of the North Sea. They also showed that the competition between temperature and biological controls can be strongly affected by riverine nutrient inputs in the study region which stimulate biological activity. Recent work in waters around Ireland showed the impact of primary productivity on carbon uptake [63], and also of riverine inputs on carbonate chemistry in near coastal waters with pronounced variability between these inputs depending on the nature of the river catchments (carbonate and silicate mineralogy or other) and land-use.

### 3.4. pH Dynamics

#### 3.4.1. Primary Production

**Figure 7:**
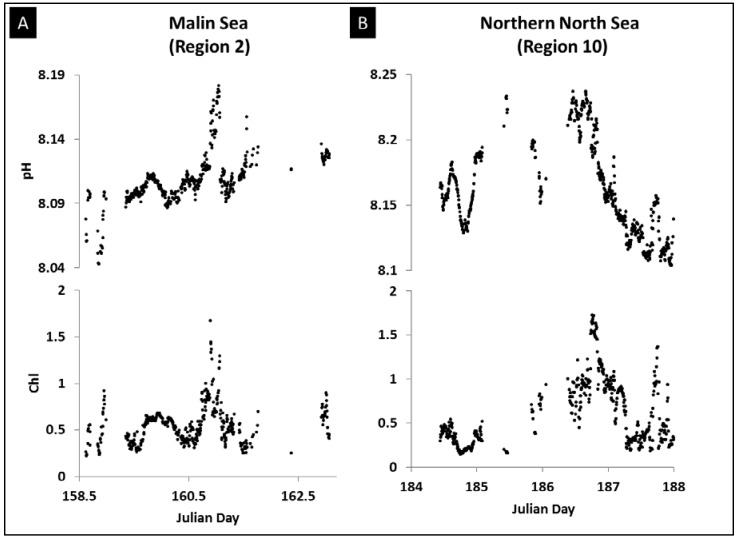
Observed sea surface pH and chlorophyll fluorescence (Chl) plotted against Julian day for (**A**) Malin Sea region of the North Atlantic Ocean west of Ireland (region 2), (**B**) Northern North Sea region (region 10).

The impact of primary production on the pH distribution is evidenced by the positive correlation between chlorophyll and pH as observed in the section of the North Atlantic Ocean to the west of Ireland (region 2, Malin) (Figure 7). However, pH is not necessarily directly correlated to the chlorophyll concentration as the stage of the phytoplankton bloom, and zooplankton grazing and remineralisation processes will influence the carbonate chemistry of waters with a phytoplankton bloom. For example, Watson and co-workers proposed three different linear relationships between pCO_2_ and chlorophyll corresponding to the different stages of development of the phytoplankton bloom [65]: the “recent history bloom”, the “peak stage” of the bloom and the “late stage” of the bloom. This is related to the fact that carbonate chemistry changes in phytoplankton blooms occur at shorter timescales than air-sea CO_2_ exchanges. 

An observed anti-correlation between nutrients and pH reinforces our point that primary production formed a major control of pH in parts of the study region. In region 10 (Northern North Sea) the multi-linear regression with SSS, SST and chlorophyll highlighted the strong correlation of chlorophyll with pH over SSS and SST as also reported by e.g., [28,60], but the regression only described 25% of the pH variance (Table 2). The positive correlation between pH and temperature in regions 1, 2, 3, 5, 7 and 10 provided a further indication that primary production was a major variable controlling the pH distribution in these regions. The positive correlation can be related to the development of phytoplankton growth in warming waters following early spring, with a minor direct contribution by temperature on primary production (μ = a*b^T^ with μ specific growth rate, a and b constants [34]).

#### 3.4.2. Temperature

**Figure 8:**
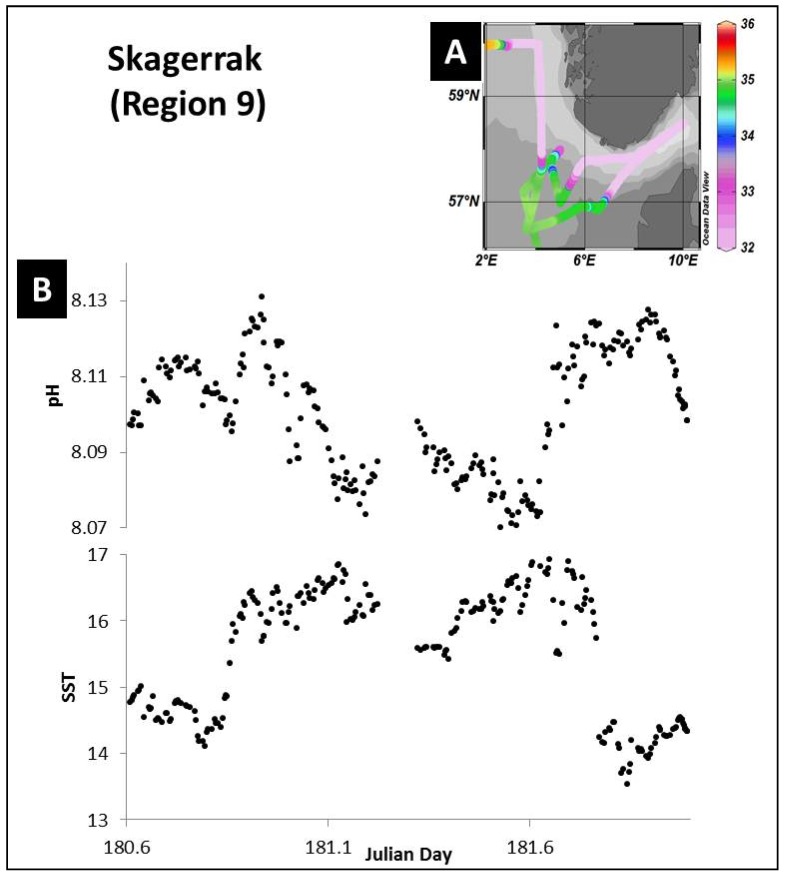
Impact of temperature on sea surface pH distributions. (**A**) Map of sea surface salinity observed during the cruise D366 in the Skagerrak region (region 9). (**B**) Sea surface pH and temperature plotted versus Julian day for the Skagerrak region. Map was produced using Ocean Data View (Schlitzer, R., Ocean Data View, odv.awi.de, 2017).

The impact of temperature on the pH distribution is evidenced here in the Skagerrak area (region 9) (Figure 8A,B). Along the Norwegian coast relatively warm low salinity (and low TA, DIC) waters of the Baltic Sea are discharged (Figure 8A). Temperature distributions explained ca. 50% of the pH variation in this region. Figure 8B shows the anti-correlation between temperature and pH with a decrease in pH as temperature increased from the North Sea into the Skagerrak Strait, as a consequence of the impact of temperature on the carbonate equilibrium constants [33]. Similar temperature driven influences on pH and pCO_2_ have been demonstrated for waters of the North Sea [66].

#### 3.4.3. Organic and Inorganic River Inputs: Remineralisation

Riverine inputs of nutrients enhance primary production (increase in pH), whereas the addition of organic matter may result in a decrease in pH through remineralisation processes with the release of CO_2_. However, in our observations we only note the resulting balance of the two processes. The DOC distribution could explain 15% of the pH variance along the full transect. In two areas, the Strait of Moyle in the Irish Sea (northern part of region 1) and Southern North Sea (region 7), the primary production was not sufficiently strong to balance the remineralisation processes with similar observations reported for summer periods [60,66]. Primary production in the Southern North Sea was likely limited by low light levels as a consequence of a fully mixed water column and an enhanced turbidity [67]. Enhanced levels of remineralisation were evidenced by the high concentrations of dissolved organic matter (DOC, dissolved organic nitrogen (DON) and phosphorus (DOP)) and low concentrations of particulate organic matter (particulate organic carbon (POC), nitrogen (PON) and phosphorus (POP)) in this region. The lower salinity (and lower TA) observed in these two regions, supports the hypothesis that the organic matter was derived from riverine inputs. Low TA resulted in a reduced buffering capacity [33] and would explain that these two regions were sources of CO_2_ to the atmosphere (see Figure 5).

#### 3.4.4. Mixing

**Figure 9:**
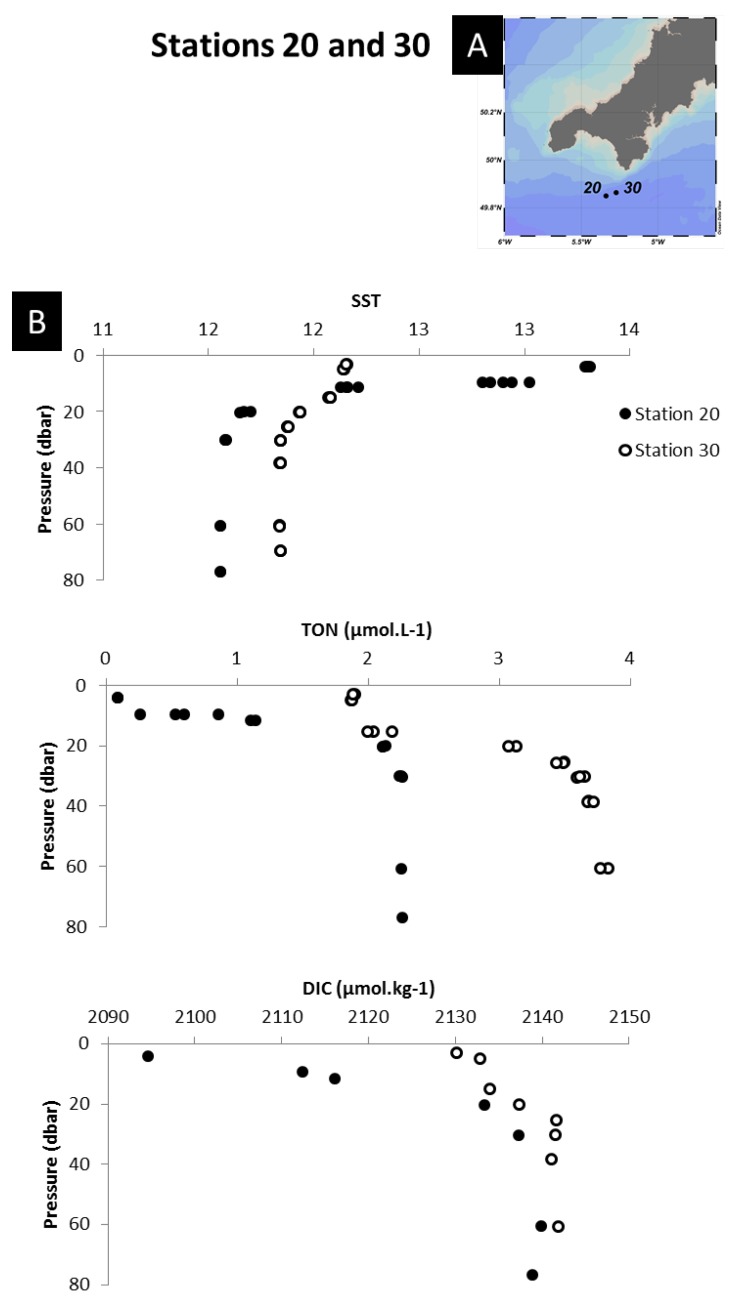
Impact of storm mixing on sea surface pH. (**A**) Location of station 20 sampled on the 15 June 2011 and station 30 sampled on the 30 June 2011 south of Cornwall (UK). (**B**) Depth profiles of temperature (SST), nitrate and nitrite (TON) and DIC at the two stations. Map was produced using Ocean Data View (Schlitzer, R., Ocean Data View, odv.awi.de, 2017).

**Figure 10:**
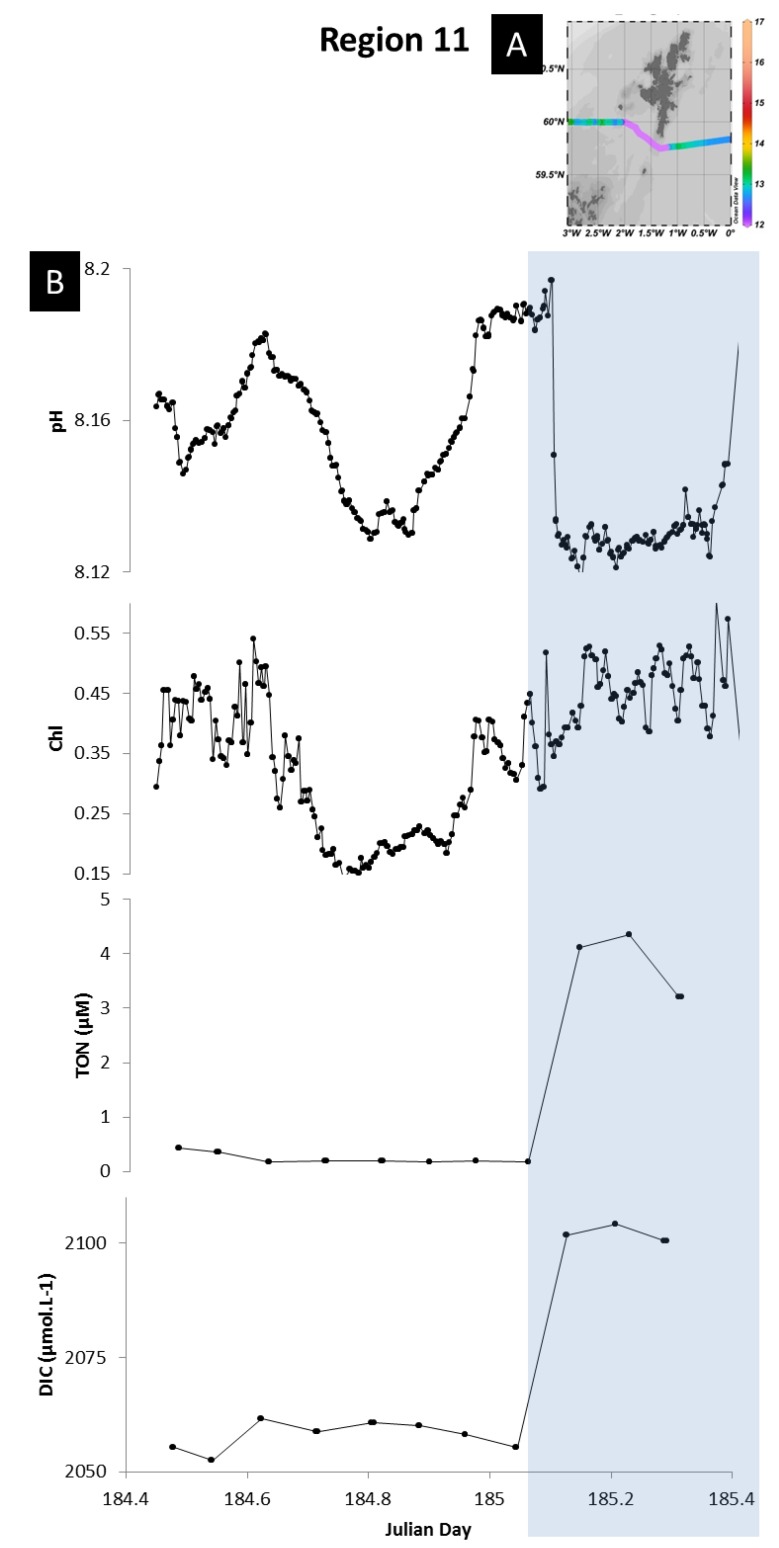
Impact of tidal shelf mixing on sea surface pH. (**A**) Sea surface temperatures observed in region south of the Orkney Islands. (**B**) Time series plots of sea surface pH, chlorophyll, DIC and Nitrate and nitrite concentrations (TON), plotted against Julian day. Map was produced using Ocean Data View (Schlitzer, R., Ocean Data View, odv.awi.de, 2017).

Tidal and wind-driven currents are important in the Northwest European shelf waters [68]. These currents can lead to intense mixing of the water column, and include tidal upwelling which brings bottom water to the surface [67]. These deeper waters are typically more acidic, enriched in DIC and nutrients relative to the surface waters due to remineralisation of organic matter. Acidic waters brought to the surface by shelf mixing processes have been observed in two regions during the cruise, and noted in our dataset due to the high resolution pH measurements. The first region was to the south of Cornwall (UK) (region 4) (Figure 9A), where the water column was likely mixed by strong tidal mixing [67]. The mixing of the water column is evidenced on Figure 9B, where the temperature gradients showed a typical stratified water column temperature profile on 15 June 2011 (red) and a more homogenised temperature profile on 23 June 2011 (green). This mixing resulted in surface waters with higher concentrations of DIC (+36 µmol kg^−1^) and TON (+2 µM), and lower pH, compared to observation made in the previous week (Figure 9C,D). The second region was south of the Shetland Islands (region 11) where deep water was brought to surface partly by the advection of Atlantic water through the Orkney-Shetland passage but also by intense tidal mixing around the Orkney and Shetland Isles area [69] (Figure 10). Indeed, tidal currents are intensified in the area where the flow is constrained by topography [54]. This upwelled water was evidenced by colder temperatures (−2 °C) and enhanced DIC (+40 µmol kg^−1^) and TON (+4 µM) concentrations compared to surrounding waters. Higher nutrient concentrations resulted in enhanced primary production but this was not sufficiently pronounced to balance the acidity of the upwelled water.

## 4. Conclusions

The successful deployment of a novel pH sensor based on Lab-on-Chip technology with spectrophotometric detection, resulted in a high quality pH data set of 29,950 samples with a precision of 0.001 pH units and an accuracy of 0.004 pH units. The sensor was deployed in an autonomous manner, requiring only infrequent calibration with reference materials as the technique is virtually calibration free. The underway operation was only interrupted infrequently in order to run reference materials. Our study presents the first comprehensive high resolution pH dataset for the Northwest European Shelf Seas, with data directly obtained from high quality spectrophotometric observations rather than values calculated from pairs of carbonate chemistry variables like TA and DIC or TA and pCO_2_. Furthermore, very few carbonate chemistry observations are available for the Celtic Sea, Malin Sea and therefore our work provides unique data obtained using novel sensor technology. The high spatial and temporal resolution of the pH data along with temperature, salinity, chlorophyll and nutrients, allowed us to unravel the main processes controlling the carbonate chemistry dynamics of the Northwest European shelf surface waters in summer. The statistical approach allowed us to determine the main correlations between pH and other variables, and infer the main controlling processes. In agreement with previous studies, photosynthesis and respiration formed the main control of the pH distribution along the cruise transect for this summer cruise. However, in some regions temperature and riverine inputs balanced or even dominated the impact of primary production. This study highlights the strong variability of the in situ conditions in coastal shelf waters where several processes simultaneously impact the carbonate chemistry. The spatial variability is particularly important in shelf seas with rivers and water column mixing providing a relatively localised impact on the seawater carbonate chemistry. Datasets with a high spatial and temporal coverage are required to allow a more accurate estimates of carbonate chemistry in coastal regions. The pH variability in coastal waters is higher than in oceanic waters [7], making it challenging to decipher the continuous decrease in pH on coastal system, caused by ocean acidification, against natural variability. Nevertheless, organisms living in waters featuring strong changes in pH are more tolerant to ocean acidification [70,71] which makes coastal ecosystems more robust. The next step in the development of the Lab-on-Chip pH sensor involves the development, optimisation and deployment of an in situ unit for autonomous deployment on platforms such as moorings, gliders and benthic landers. The first steps have been made, and we will report on validation and optimisation of the instrument in the near future

## Figures and Tables

**Table 1:** Mean values and standard deviations (Std; 1δ) of S, T, TON, SiO4 and Chl per regions. Irish Sea (region 1), Malin Sea; northwest of Ireland (region 2), Celtic Sea (region 3), south of Cornwall, UK (region 4), Bay of Biscay (region 5), English Channel (region 6), Southern North Sea (region 7), Central North Sea (region 8), Skagerrak (region 9), Northern North Sea (region 10), South of the Shetland Islands (region 11).

Region	Mean SSS	Std SSS (PSU)	Mean SST (°C)	Std SST (°C)	Mean NO_3_ (μM)	Std NO_3_ (μM)	Mean PO_4_ (μM)	Std PO_4_ (μM)	Mean SiO_2_ (μM)	Std SiO_2_ (μM)	Mean Chl (µg L^−1^)	Std Chl (µg L^−1^)
1	34.11	0.23	11.06	0.60	1.32	1.15	0.24	0.10	1.51	0.93	0.30	0.11
2	35.14	0.23	11.34	0.11	3.15	1.71	0.21	0.10	2.00	0.64	0.59	0.24
3	35.24	0.14	13.63	0.40	0.10	0.00	0.06	0.06	1.12	0.92	0.20	0.07
4	35.38	0.06	13.59	0.94	0.63	0.92	0.06	0.04	0.58	0.54	0.39	0.20
5	35.65	0.10	14.55	0.59	0.80	0.64	0.08	0.04	1.06	0.24	0.43	0.27
6	35.09	0.11	14.09	0.29	0.90	0.74	0.06	0.03	1.97	0.41	0.38	0.09
7	34.28	0.44	14.99	0.65	1.07	1.58	0.07	0.05	1.56	1.34	0.31	0.16
8	34.86	0.14	13.68	0.26	0.17	0.03	0.04	0.08	0.42	0.33	0.20	0.05
9	30.88	2.09	14.69	1.24	0.18	0.06	0.03	0.03	0.15	0.10	0.30	0.07
10	35.25	0.16	12.50	0.31	0.55	0.71	0.06	0.05	0.44	0.29	0.63	0.41
11	35.31	0.01	10.95	0.10	3.89	0.54	0.33	0.04	1.61	0.44	0.43	0.04

**Table 2:** Results of the multi-linear regression analysis using SSS, SST and Chl variables with the underway data divided into 11 regions. The R^2^ values express how much of the pH variance is explained by the regression. The component of the pH variance that can be attributed to each variable is expressed in percentage. The SSS, SST amd Chl values in the lower part of the panel detail the equation coefficients of each parameter used to describe pH. Irish Sea (region 1), Malin Sea; northwest of Ireland (region 2), Celtic Sea (region 3), south of Cornwall, UK (region 4), Bay of Biscay (region 5), English Channel (region 6), Southern North Sea (region 7), Central North Sea (region 8), Skagerrak (region 9), Northern North Sea (region 10).

Regions	R1	R2	R3	R5	R6	R7	R8	R9	R10
R^2^	0.79	0.40	0.37	0.58	0.41	0.21	0.65	0.63	0.25
SSS	5.91	36.65	28.85	31.81	54.67	30.06	6.53	19.99	8.55
SST	45.62	12.12	37.26	35.50	29.06	16.41	50.99	73.01	6.11
Chl	48.47	51.23	33.89	32.69	16.27	53.53	42.47	7.00	85.34
Intercept	8.080	8.107	8.112	8.111	8.078	8.080	8.094	8.111	8.161
SSS	0.002	−0.007	0.010	0.007	0.011	0.005	−0.001	0.005	0.002
SST	0.014	0.002	0.013	0.008	−0.006	0.003	−0.011	−0.017	0.001
Chl	0.015	0.009	0.012	0.007	−0.003	0.010	0.009	0.002	0.018
